# Evaluation of Personal Protective Equipment Usage Among Construction Workers in Erbil City, Iraq

**DOI:** 10.7759/cureus.68937

**Published:** 2024-09-08

**Authors:** Salih Ahmed Abdulla

**Affiliations:** 1 Department of Community Nursing, College of Nursing, Hawler Medical University, Erbil, IRQ

**Keywords:** construction workers, erbil city, occupational safety, personal protective equipment (ppe), workplace compliance

## Abstract

Background and aim

The construction industry is a high-risk environment where the use of personal protective equipment (PPE) is essential for worker safety. Despite the clear benefits of PPE, compliance rates among construction workers are often suboptimal. In response to these concerns, this study aimed to assess the knowledge and attitudes regarding PPE usage among construction workers in Erbil City, Iraq.

Methods

This cross-sectional study was conducted from December 15, 2022, to June 15, 2023, among construction workers in Erbil. Convenience sampling was used to collect data through a self-structured questionnaire. The questionnaire collected demographic information, as well as responses to a 15-item Knowledge Questionnaire and an 8-item Attitude Questionnaire. Statistical analysis was performed using IBM SPSS Statistics for Windows, Version 28 (Released 2021; IBM Corp., Armonk, New York). Frequency and percentage were used to describe qualitative variables, while mean and standard deviation were calculated for quantitative variables. Parametric tests such as the independent sample t-test and ANOVA were used, along with multinomial logistic regression, to assess the relationships between knowledge, attitude, and various demographic factors.

Results

A total of 280 workers participated in the study. The mean knowledge score was 13.56 ± 1.17, indicating a good level of knowledge, while the mean attitude score was 6.86 ± 1.11, reflecting a fair attitude toward PPE usage. A significant majority of the participants, 97.9% (274), were categorized as having good knowledge, while 68.2% (191) exhibited a fair attitude towards PPE usage. The analysis showed that demographic factors such as age, marital status, working hours, work experience, and employment type did not significantly affect knowledge or attitude, with all odds ratios (ORs) near 1 and P-values above 0.05.

Conclusion

The findings indicate that construction workers in Erbil generally have good knowledge but only a fair attitude toward PPE usage. These results suggest that healthcare providers and policymakers should implement targeted educational interventions to improve workers' attitudes toward PPE, aiming to boost compliance and enhance workplace safety. Additionally, these interventions should address practical barriers to PPE usage, such as discomfort or lack of accessibility. By fostering more positive attitudes and ensuring the availability of necessary resources, overall safety in the construction industry can be significantly improved.

## Introduction

Personal protective equipment (PPE) is specialized gear designed to safeguard workers from health and safety risks associated with their jobs, particularly in the construction sector [1-3]. PPE includes hard hats, safety glasses, gloves, and high-visibility clothing, which are designed to protect workers from potential hazards and injuries on the job site [4]. Given the high-risk nature of construction work, the construction industry is known for its hazardous environment, with workers exposed to a wide range of dangers, including falls, electrical hazards, and exposure to harmful substances [5]. Nevertheless, despite the clear benefits of PPE, studies have shown that compliance with PPE usage among construction workers is often suboptimal, with compliance rates ranging from 50% to 75% in different settings [6].

Understanding the factors that affect compliance is essential. The knowledge and attitude of construction workers regarding PPE usage are crucial factors influencing their compliance and overall safety on the job site. Knowledge refers to the understanding and awareness of the importance, proper use, and maintenance of PPE [7]. Workers who understand the benefits and limitations of PPE are more likely to use it consistently and effectively [8]. On the other hand, inadequate knowledge can lead to improper use or non-use of PPE, increasing the risk of accidents and injuries, with studies showing that 20% to 40% of injuries in construction workers could be attributed to improper or non-use of PPE [9].

In addition to knowledge, attitude plays a critical role. Attitude, which encompasses beliefs, feelings, and behavioral tendencies toward PPE usage, also plays a significant role in workers' safety practices [10]. A positive attitude towards PPE is associated with higher compliance and a proactive approach to safety, while a negative attitude can lead to resistance and non-compliance [11]. Factors influencing workers' attitudes include perceived comfort, fit, and effectiveness of PPE, as well as the safety culture within the organization [12]. This is particularly relevant in Erbil City, the capital of the Kurdistan Region of Iraq, which has experienced significant growth in recent years, driven by increased infrastructure development and urban expansion [13]. However, this growth has also brought challenges in ensuring the safety and well-being of construction workers. Studies conducted in other regions of Iraq have highlighted deficiencies in PPE usage and safety practices among construction workers [14,15]. Therefore, these findings underscore the need for a comprehensive understanding of the knowledge and attitudes of construction workers in Erbil regarding PPE usage.

Several factors can influence the knowledge and attitudes of construction workers regarding PPE usage. Education level and safety training have been found to have a positive impact on workers' understanding and compliance with safety practices [16]. Workers with higher levels of education and those who have received adequate safety training are more likely to recognize the importance of PPE and use it consistently [8]. Additionally, the safety culture within the organization, including management commitment to safety and the availability of PPE, can significantly influence workers' attitudes and behaviors [17]. The implications of non-compliance are severe, as the consequences of inadequate knowledge and negative attitudes regarding PPE usage can be serious. Workers who do not use PPE properly or consistently are at a higher risk of accidents, injuries, and occupational diseases [9]. These incidents not only affect the health and well-being of the workers but also result in lost productivity, increased healthcare costs, and legal liabilities for the organization [18]. Furthermore, the lack of PPE usage can contribute to the overall poor safety performance of the construction industry, which has been a long-standing concern globally [10].

Despite the importance of understanding the knowledge and attitudes of construction workers regarding PPE usage, research on this topic in the context of Erbil City is scarce. While studies have been conducted in other regions of Iraq and globally, the unique cultural, socioeconomic, and regulatory factors in Erbil warrant a specific investigation. Moreover, the rapid growth of the construction industry in Erbil in recent years necessitates an up-to-date assessment of workers' knowledge and attitudes to inform targeted interventions and policy decisions. Thus, the present study aims to investigate the knowledge and attitudes regarding personal protective equipment usage among construction workers in Erbil City.

## Materials and methods

Study design, setting, and period

This was a cross-sectional study conducted in Erbil among workers who work in various buildings. The convenience sampling method was used to collect data from December 15, 2022, to June 15, 2023.

Sample size

The sample size was calculated using the infinite population sample size formula. We considered a 95% confidence interval, a margin of error of 5.86%, a population proportion of 50%, and a Z score of 1.96. The final sample size was 280 participants.

Inclusion/exclusion

The study's inclusion criteria included male workers aged 18 years and above who had been employed for at least one year before the date of research. Exclusion criteria consisted of workers who were absent, on leave, or on night duty during the study period, as well as those who had worked for less than one year and female workers.

Study tools and data collection

The questionnaire was divided into three main sections. The first section collected demographic information, including age, marital status, working hours, work experience, and employment type. The second section comprised a self-administered questionnaire with 15 items designed to assess the workers' knowledge levels. The third section included an 8-item self-administered questionnaire aimed at evaluating the workers' attitudes toward occupational safety and health practices. To ensure the accuracy of the data, the questions were translated from English to Kurdish using a forward-backward translation method, and the translation was verified by specialists in the field. The questionnaires were distributed to participants who met the inclusion criteria, with each participant given 10 to 15 minutes to complete it. For those unable to read, assistance was provided by the researcher to ensure accurate completion of the questionnaire.

Pilot study

The study's structured questionnaires, designed to assess knowledge and attitude, were initially tested in a pilot study involving 25 workers. The pilot study was conducted between December 1, 2022, and November 1, 2022. The internal consistency and reliability of the questionnaire items were evaluated using Cronbach's alpha [19]. The knowledge questionnaire yielded an overall Cronbach's alpha of 0.82, indicating a very good level of reliability. Similarly, the attitude questionnaire, tested on the same group of 25 workers, resulted in an overall Cronbach's alpha of 0.79, reflecting good reliability. To further ensure the validity of the instruments, the questionnaires were reviewed by 10 nursing professors across different specialties. It is important to note that the data from this pilot study were excluded from the final analysis.

Measures

Sociodemographic Characteristics

The first section of the questionnaire included sociodemographic information of the workers, such as age, marital status, working hours, working experience, and employment type.

Knowledge Questionnaire

The second part of the questionnaire was a self-structured Knowledge Questionnaire designed to assess workers' understanding of relevant workplace policies, safety protocols, and general occupational health awareness. This instrument comprised 15 items, with each item being a dichotomous question, where the responses were either "No" (scored as 0) or "Yes" (scored as 1). The total score ranged from 0 to 15. The scoring was categorized as follows: a score of 1-5 was considered poor knowledge, 6-10 was classified as fair knowledge, and 11-15 was categorized as good knowledge. The internal consistency of the Knowledge Questionnaire was assessed using Cronbach's alpha [17], which showed an overall reliability score of 0.82, indicating a very good level of reliability.

Attitude Questionnaire

The third part of the questionnaire was a self-structured Attitude Questionnaire designed to assess workers' perceptions and attitudes toward occupational safety and health practices. This instrument comprised eight items, with each item being a dichotomous question, where the responses were either "No" (scored as 0) or "Yes" (scored as 1). The total score ranged from 0 to 8. The scoring was categorized as follows: a score of 1-3 was considered poor attitude, 4-6 was classified as fair attitude, and 7-8 was categorized as good attitude. The internal consistency of the Attitude Questionnaire was assessed using Cronbach's alpha [17], which showed an overall reliability score of 0.79, indicating good reliability.

Ethical approval and informed consent

This study followed the Institutional Research Ethics Board and the Declaration of Helsinki guidelines. The study was approved by the Hawler Medical University College of Nursing's Ethics Committee (Approval No. 2237) on November 19, 2022. Oral informed consent was obtained from all participants before they filled out the questionnaires.

Statistical analysis

Data were summarized and reported using frequency and percentage for qualitative variables. Quantitative variables with a normal distribution were presented as mean and standard deviation. Since the data were normally distributed, parametric tests such as the independent sample t-test and ANOVA were used to assess the relationship between knowledge, attitude, and confounding variables. To further evaluate these relationships, multinomial logistic regression was employed. Data analysis was performed using IBM SPSS Statistics for Windows, Version 28 (Released 2021; IBM Corp., Armonk, New York), with significance levels set at P < 0.05.

## Results

Demographic characteristics

A total of 280 workers participated. The participants' ages ranged from 18 to over 50 years, with a mean age of 32.45 ± 8.44 years. The majority of participants were in the 30-39 age group, comprising 124 (44.3%) of the sample, while those aged 20-29 accounted for 89 (31.8%). Most participants were married, totaling 200 (71.4%), with the remaining 80 (28.6%) being single. Regarding working hours, 176 (62.9%) of participants reported working 7-8 hours daily. In terms of work experience, 124 (44.3%) had 6-10 years of experience, and 77 (27.5%) had 1-5 years. The majority of participants, 252 (90.0%), were employed on a temporary or daily basis, while only 28 (10.0%) were permanent employees. Knowledge levels were predominantly high, with 274 (97.9%) classified as having good knowledge, reflected by a mean score of 13.56 ± 1.17. Attitude levels were generally fair, with 191 (68.2%) demonstrating a fair attitude (mean score: 6.86 ± 1.11). For further details, refer to Table 1.

**Table 1:** Demographic Characteristics, Work Conditions, and Knowledge and Attitude Levels Among Construction Workers in Erbil City (N = 280) Results are expressed as N (%) or mean ± SD.

Items	Categories (n=280)	N	%
Age (year)	Less than 20	9	3.2
	20-29	89	31.8
	30-39	124	44.3
	40-49	50	17.9
	50 and above	8	2.9
	Mean ± SD	32.45 ± 8.44	
Marital status	Single	80	28.6
	Married	200	71.4
Working hours	Less than 5 hours	33	11.8
	5-6 hours	43	15.4
	7-8 hours	176	62.9
	More than 8 hours	28	10.0
Working experience	Less than 1 year	21	7.5
	1-5 years	77	27.5
	6-10 years	124	44.3
	More than 10 years	58	20.7
Employment type	Permanent	28	10.0
	Temporary/daily	252	90.0
Knowledge levels	Poor	1	0.4
	Fair	5	1.8
	Good	274	97.9
	Mean ± SD	13.56 ± 1.17	
Attitude levels	Poor	1	0.4
	Fair	88	31.4
	Good	191	68.2
	Mean ± SD	6.86 ± 1.11	

Knowledge assessment

The participants demonstrated a high awareness of PPE and its benefits. A significant majority, 267 (95.4%), had heard of PPE, with a mean score (MS) of 0.95. Additionally, 269 (96.1%) believed that PPE could protect against insect bites, the highest awareness level observed (MS = 0.96). Similarly, 266 (95.0%) recognized that PPE could prevent hand injuries and protect against cough (MS = 0.95 for both). Awareness was slightly lower, though still strong, regarding the protection PPE offers against lung problems, with 214 (76.4%) acknowledging the benefits of respiratory masks (MS = 0.76). Overall, the data indicate a solid understanding among participants of the protective benefits of PPE in various aspects of workplace safety. For further details, refer to Table 2.

**Table 2:** Participants' Knowledge Regarding the Effectiveness and Importance of PPE in the Workplace (N = 280) N is the total number of participants in the study; results are expressed as N (%) or mean score (MS).

Items	Scales		
	Yes, n (%)	No, n (%)	Mean score
PPE (personal protective equipment) includes items like helmets, gloves, and safety boots.	267 (95.4)	13 (4.6)	0.95
Excessive noise exposure can damage hearing.	253 (90.4)	27 (9.6)	0.90
Inhaling dust can cause respiratory sickness.	237 (84.6)	43 (15.4)	0.85
Workplaces with heavy machinery and construction activities are considered dangerous.	237 (84.6)	43 (15.4)	0.85
Safety boots are designed to protect against foot injuries	252 (90.0)	28 (10.0)	0.90
Respiratory masks are effective in protecting against lung problems.	214 (76.4)	66 (23.6)	0.76
Ear protection can prevent hearing loss.	257 (91.8)	23 (8.2)	0.92
Overalls are used to protect against body injuries at the workplace.	240 (85.7)	40 (14.3)	0.86
Helmets are worn to protect from head injuries.	262 (93.6)	18 (6.4)	0.94
Eye protection is essential to prevent eye injuries.	262 (93.6)	18 (6.4)	0.94
Protective gloves are effective in preventing hand injuries.	266 (95.0)	14 (5.0)	0.95
Using PPE can prevent injuries in the workplace.	264 (94.3)	16 (5.7)	0.94
PPE can protect against insect bites.	269 (96.1)	11 (3.9)	0.96
Using PPE can help protect against coughs.	266 (95.0)	14 (5.0)	0.95
Using PPE can protect against various diseases.	252 (90.0)	28 (10.0)	0.90

Attitude assessment

The data revealed strong positive attitudes toward the importance and use of PPE in the workplace. A significant proportion of participants, 231 (82.5%), believe that PPE is crucial for ensuring workplace safety, with a mean score (MS) of 0.83. Additionally, 247 (88.2%) agree that employees who do not use PPE should be punished (MS = 0.88), and 244 (87.1%) think it should be mandatory for all employees to use PPE (MS = 0.87). The majority, 252 (90.0%), feel that PPE is absolutely necessary in their workplace (MS = 0.90), while 238 (85.0%) acknowledge that PPE can be bothersome during work (MS = 0.85). However, 232 (82.9%) disagree that PPE is a waste of money (MS = 0.83). Overall, the results indicate a high level of recognition of the importance of PPE among participants, despite some discomfort associated with its use. For further details, refer to Table 3.

**Table 3:** Attitudes of Participants Toward the Use and Enforcement of PPE in the Workplace (N = 280) N is the total number of participants in the study; results are expressed as N (%) or mean score (MS).

Items	Scales		
	Yes, n (%)	No, n (%)	Mean score
Do you believe that PPE (personal protective equipment) is important for ensuring workplace safety?	231 (82.5)	49 (17.5)	0.83
Should employees who do not use PPE be punished?	247 (88.2)	33 (11.8)	0.88
Do you think it should be compulsory for all employees to use PPE?	244 (87.1)	36 (12.9)	0.87
Should employees be allowed to decide for themselves whether to use PPE?	243 (86.8)	37 (13.2)	0.87
Do you believe that the PPE provided in your work area is of good quality?	235 (83.9)	45 (16.1)	0.84
Is the use of PPE necessary in your workplace?	252 (90.0)	28 (10.0)	0.90
Does PPE bother you while you are working?	238 (85.0)	42 (15.0)	0.85
Do you think PPE is a waste of money?	232 (82.9)	48 (17.1)	0.83

Knowledge and attitude by demographic and work variables

The analysis showed that knowledge scores were relatively consistent across different age groups, with the MS ranging from 13.46 to 14.33, and no significant differences were observed (P > 0.05). Attitude scores were also similar across age groups, with the highest mean score of 6.89 in the 20-29 age group. Marital status did not significantly influence knowledge or attitude, as both single and married participants exhibited nearly identical scores (P > 0.05). Working hours and experience also showed no substantial impact on either knowledge or attitude, with mean scores remaining stable across various categories. Employment type revealed slightly lower knowledge scores among permanent employees (MS = 13.39) compared to temporary or daily workers (MS = 13.58), though this difference was not statistically significant (P > 0.05). Similarly, attitude scores were consistent across employment types. Please see Table 4 for additional details.

**Table 4:** Factors Associated With Knowledge and Attitude Scores Among Construction Workers by Demographic and Professional Characteristics (N = 280) ^a^Independent sample t-test (T) was used to assess the relationship between knowledge and attitude with demographic data. ^b^One-way ANOVA test (F) was used to assess the relationship between knowledge and attitude with demographic data. N is the total number of participants in the study; results are expressed as N (%) or mean score (MS). Significance was set at P < 0.05.

			Knowledge				Attitude			
Variables	Items	Number	MS	SD	T\F	P-value	MS	SD	T\F	P-Value
Age (year)	Less than 20	9	14.33	0.71	1.29^b^	0.28	6.78	1.20	0.24^b^	0.91
	20-29	89	13.47	1.26			6.89	1.04		
	30-39	124	13.61	1.15			6.88	1.13		
	40-49	50	13.46	1.03			6.86	1.16		
	50 and above	8	13.63	1.30			6.50	1.20		
Marital status	Single	80	13.53	1.12	0.39^a^	0.72	6.91	1.09	0.41^a^	0.65
	Married	200	13.58	1.18			6.85	1.12		
Working hours	Less than 5 hours	33	13.45	1.30	0.26^b^	0.85	6.91	1.13	0.36^b^	0.78
	5-6 hours	43	13.53	1.16			6.81	1.10		
	7-8 hours	176	13.57	1.17			6.90	1.11		
	More than 8 hours	28	13.71	1.01			6.68	1.16		
Working experience	Less than 1 year	21	13.62	1.12	0.20^b^	0.90	7.00	1.10	0.17^b^	0.92
	1-5 years	77	13.53	1.28			6.84	1.04		
	6-10 years	124	13.61	1.15			6.88	1.14		
	More than 10 years	58	13.48	1.06			6.81	1.16		
Employment type	Permanent	28	13.39	1.13	0.02^a^	0.87	6.96	1.10	1.05^a^	0.31
	Temporary/daily	252	13.58	1.17			6.85	1.11		

Regression correlations with demographic data

The multinomial logistic regression analysis indicated that demographic and work-related factors such as age, marital status, working hours, work experience, and employment type did not significantly influence either knowledge or attitude among the participants. All odds ratios (ORs) were close to 1, and the P-values were above 0.05, indicating no substantial differences across these variables. For example, participants under 20 years had an OR of 2.04 (95% CI: 0.76-5.50, P = 0.16) for knowledge, and single participants had an OR of 0.96 (95% CI: 0.77-1.20, P = 0.71) for knowledge, neither of which was statistically significant. Similarly, employment type showed no significant association, with permanent workers having an OR of 0.87 (95% CI: 0.63-1.21, P = 0.40) for knowledge. These results suggest that none of these factors had a meaningful impact on the knowledge or attitude of the participants. For a more detailed breakdown, please refer to Table 5.

**Table 5:** Multinomial Logistic Regression Analysis of Demographic and Professional Factors Predicting Knowledge and Attitude Scores Among Nurses (N = 280) N is the total number of participants in the study; multinomial logistic regression analysis was used. Significance was set at P < 0.05. OR: odds ratio, CI: confidence interval, LB: lower band, UB: upper band, Ref: reference.

Items	Knowledge				Attitude			
	OR	95% CI		P-Value	OR	95% CI		P-Value
		LB	UB			LB	UB	
Age (50 and above is the ref.)								
Less than 20 years	2.04	0.76	5.50	0.16	1.22	0.54	2.77	0.63
20-29 years	0.89	0.74	1.68	0.72	1.33	0.73	2.45	0.35
30-39 years	0.99	0.53	1.86	0.98	1.32	0.73	2.42	0.34
40-49 years	0.86	0.46	1.70	0.71	1.30	0.70	2.45	0.40
Marital status (Married is the ref.)								
Single	0.96	0.77	1.20	0.71	1.06	0.84	1.34	0.64
Working hours (More than 8 hours is the ref.)								
Less than 5 hours	0.82	0.53	1.27	0.37	1.20	0.77	1.88	0.42
5-6 hours	0.87	0.58	1.32	0.52	1.11	0.73	1.68	0.62
7-8 hours	0.89	0.63	1.27	0.53	1.19	0.84	1.68	0.33
Working experience (more than 10 years is the ref.)								
Less than 1 year	1.10	0.72	1.70	0.66	1.17	0.73	1.87	0.51
1-5 years	1.04	1.77	1.38	0.81	1.03	0.76	1.39	0.87
6-10 years	1.10	0.84	1.44	0.49	1.06	0.80	1.40	0.71
Employment type (Temporary/Daily is the ref.)								
Permanent	0.87	0.63	1.21	0.40	1.10	0.76	1.59	0.60

## Discussion

The present study aimed to investigate the knowledge and attitudes regarding PPE usage among construction workers in Erbil City, Iraq. The results demonstrated that construction workers in Erbil have good knowledge and a fair attitude towards PPE usage, reflecting a generally positive outlook on workplace safety measures. This positive outlook is particularly important given the inherent risks associated with construction work.

Construction work is inherently high-risk, with workers regularly exposed to hazards that can result in serious injuries or long-term health issues [20]. In Erbil, the rapid growth of the construction industry has emphasized the need to ensure workers are adequately protected. Despite this, there was a notable gap in the literature concerning the knowledge and attitudes of construction workers in this region toward PPE prior to this study. To address these gaps, our study specifically aimed to assess the knowledge and attitudes regarding PPE usage among construction workers in Erbil, providing insights that can inform future safety practices.

The demographic profile of our study participants, representing a diverse range of ages and marital statuses, reflects a typical distribution within the construction industry. This demographic diversity is key to understanding the varying perspectives on PPE usage. The prevalence of temporary or daily employment among workers aligns with global trends in the construction sector, where casual labor is common [21]. This trend is particularly relevant in developing regions, where job security is often limited, and the workforce is highly transient. The significant experience reported by most workers in our study suggests that these individuals are well-versed in the demands and risks associated with construction work, making their insights into PPE usage particularly valuable.

The strong awareness of PPE and its benefits among participants is a positive finding, indicating that workers in Erbil are well-informed about necessary protective measures. This high level of awareness may be attributed to the regulatory and cultural context of Iraq, where safety practices are increasingly emphasized. This knowledge level is consistent with studies conducted in other developing countries, such as Nigeria and Uganda [22,23]. Furthermore, the positive attitudes toward PPE, as evidenced by most participants recognizing its importance for workplace safety, suggest a strong foundation for improving compliance with safety protocols. However, the discomfort associated with PPE, as reported by some workers, highlights an area for improvement. Ergonomic enhancements in PPE design could play a crucial role in increasing both comfort and compliance, thereby reducing injury risks.

Interestingly, our study found that demographic factors such as age and marital status did not significantly impact knowledge or attitudes toward PPE. This uniformity across demographic groups suggests that the construction industry in Erbil may have successfully standardized safety training and awareness. This finding contrasts with some previous studies that reported these factors as influencing PPE-related behaviors [7,24]. The consistent knowledge and attitude scores across different demographic groups in our study suggest that the construction industry in Erbil may foster a standardized understanding of PPE, potentially due to the widespread implementation of safety training programs or the influence of industry-wide safety regulations.

The regression analysis revealed that work-related factors, including working hours, work experience, and employment type, did not significantly influence knowledge or attitudes towards PPE. This lack of significant influence may be due to the homogeneous safety culture in Erbil's construction sector. This result is somewhat unexpected, given that previous research identified these factors as critical determinants of PPE compliance and perceptions [25,26]. The absence of significant associations in our study may be attributed to the uniform nature of the construction workforce in Erbil, where the prevalence of temporary and daily employment creates a consistent safety culture across work settings. This finding suggests that interventions aimed at improving PPE usage may need to be tailored more to individual attitudes and organizational policies rather than relying solely on demographic or work-related factors.

Despite the valuable insights provided by this study, several limitations should be acknowledged. The cross-sectional design restricts the ability to infer causality between the studied variables, limiting our understanding of the temporal relationships involved. Moreover, the study's focus on a specific city may constrain the generalizability of the findings to other regions in Iraq or similar contexts globally. Another limitation is the absence of data on actual PPE compliance rates, which could have provided a more comprehensive view of PPE usage in practice. To address these limitations, future research should consider employing longitudinal designs, expanding the geographic scope, and incorporating direct measures of PPE compliance. Additionally, qualitative research could offer deeper insights into the personal experiences and challenges workers face regarding PPE usage, potentially uncovering new strategies for enhancing safety practices in high-risk environments like the construction industry.

## Conclusions

The results indicate that construction workers in Erbil possess good knowledge and a fair attitude regarding PPE usage. Based on these findings, it is recommended that healthcare providers and policymakers implement targeted educational interventions to improve workers' attitudes toward PPE, leading to better compliance and enhanced workplace safety. Future research should focus on identifying the specific barriers that prevent workers from fully adopting PPE and exploring the influence of organizational safety culture on compliance. Additionally, longitudinal studies could provide insights into how ongoing educational programs affect knowledge and attitudes over time. Expanding the research to include different regions and industries would also help generalize these findings and inform broader safety policies.
